# Advances in the Management of Acute Postsurgical Pain: A Review

**DOI:** 10.7759/cureus.42974

**Published:** 2023-08-04

**Authors:** Antonella Paladini, Narinder Rawal, Miquel Coca Martinez, Mehdi Trifa, Antonio Montero, Joseph Pergolizzi, Alberto Pasqualucci, Marco Antonio Narvaez Tamayo, Giustino Varrassi, Oscar De Leon Casasola

**Affiliations:** 1 Department of Life, Health and Environmental Sciences (MESVA), University of L'Aquila, L'Aquila, ITA; 2 Anesthesia, Orebro Medical School, Orebro, SWE; 3 Anesthesia, McGill University, Montreal, CAN; 4 Anesthesia, University of Tunis, Tunis, TUN; 5 Surgery, University of Lleida, Lleida, ESP; 6 Operations, Nema Research, Inc., Naples, USA; 7 Anesthesia and Critical Care, University of Perugia, Perugia, ITA; 8 Pain Medicine Unit, Hospital Obrero, La Paz, BOL; 9 Pain Medicine, Paolo Procacci Foundation, Rome, ITA; 10 Anesthesiology, Buffalo School of Medicine, Buffalo, USA

**Keywords:** prehabilitation, adjuvant analgesics, opioid analgesics, nsaids, acute pain, acute postoperative pain

## Abstract

Despite the millions of surgeries performed every year around the world, postoperative pain remains prevalent and is often addressed with inadequate or suboptimal treatments. Chronic postsurgical pain is surprisingly prevalent, and its rate varies with the type of surgery, as well as with certain patient characteristics. Thus, better clinical training is needed as well as patient education. As pain can be caused by more than one mechanism, multimodal or balanced postsurgical analgesia is appropriate. Pharmacological agents such as opioid and nonopioid pain relievers, as well as adjuvants and nonpharmacologic approaches, can be combined to provide better and opioid-sparing pain relief. Many specialty societies have guidelines for postoperative pain management that emphasize multimodal postoperative analgesia. These guidelines are particularly helpful when dealing with special populations such as pregnant patients or infants and children. Pediatric pain control, in particular, can be challenging as patients may be unable to communicate their pain levels. A variety of validated assessment tools are available for diagnosis. Related to therapy, most guidelines agree on the fact that codeine should be used with extreme caution in pediatric patients as some may be “rapid metabolizers” and its use may be life-threatening. Prehabilitation is a preoperative approach that prepares patients in advance of elective surgery with conditioning exercises and other interventions to optimize their health. Prehabilitation may have aerobic, strength-training, nutritional, and counseling components. Logistical considerations and degree of patient adherence represent barriers to effective prehabilitation programs. Notwithstanding all this, acute postoperative pain represents a clinical challenge that has not yet been well addressed.

## Introduction and background

Approximately 80% of those who undergo surgical interventions report acute postoperative pain and, of that population, 75% state that pain intensity is moderate to severe or worse [1]. The armamentarium for managing pain is large and growing, but many clinicians are unsure how to use these pharmacologic and device-based interventions to the best advantage. Acute pain should be treated, even when the case is complicated, such as postoperative pain in neonates and pain in very old and multimorbid patients. While clinicians may benefit from many guidelines about how to use pain medicine best, there is an inherently subjective and highly personal aspect to pain, such that sound clinical judgment is often needed to prescribe the optimal pain management protocol for an individual patient [1]. Clinicians must not only be alert to the signs and symptoms of postoperative pain they must be knowledgeable about the analgesic armamentarium. This paper aims to summarize the findings of an expert panel on acute postoperative pain control and the implications for pain medicine.

## Review

Methodology

This paper is a narrative review based on presentations by the authors at the second annual meeting of “Past, Present, and Future in Pain Medicine” held in Tunis, Tunisia, on May 11 to 13, 2023. It is a narrative review with clinical observations from the speakers/authors.

Results

Better Management of Acute Postoperative Pain

Postoperative pain may be counted among the most concerning of the many worries preoccupying preoperative patients, with about half of the patients awaiting elective surgery stating they suffered “high” or “very high” anxiety about the pain they might experience after the operation [2]. Such fears are not unrealistic; on a survey of 300 surgical patients, 86% had postoperative pain, and 75% reported experiencing postoperative pain intensities at levels they described as moderate to extreme [2]. The majority of patients (88%) received analgesics to manage postoperative pain and, of that population, 80% stated they experienced some adverse effects. Thus, postsurgical pain control often ranges from suboptimal to inadequate, and this problem of unalleviated pain is particularly severe in developing nations [3,4].

Of course, there is no one-size-fits-all postoperative pain protocol. Specific groups of patients pose a particular challenge in pain management, such as patients who are pregnant, geriatric, neonatal, and opioid-tolerant patients, as well as those with hepatic/renal disease or active substance use disorder [4]. Ambulatory surgery also presents specific challenges for postsurgical pain control, as much of the postoperative pain trajectory is managed in the home setting [5]. Physicians’ background and analgesic strategies may also vary by patient’s conditions, comorbidities, type of surgery, and rehabilitation.

The incidence of chronic postsurgical pain (CPSP) varies by type of surgery, but is surprisingly common, ranging from 10% to 70% [6,7]. In some instances, CPSP may cause not only pain but also loss of function, disability, and distress [8,9]. CPSP rates are 50% for patients undergoing thoracotomy, mastectomy, and limb amputation. Whereas patients undergoing hip and knee arthroplasty may experience it in 30-45% of the cases [9]. Children may be particularly vulnerable to CPSP, but there are few studies despite the relatively large number of surgeries performed in this population [10]. Risk factors for CPSP include patient-specific factors, such as genetic predisposition and individual psychology; surgery-specific factors; and social determinants, such as low income or poor education [11,12]. Further research is required to develop a validated risk model that would help stratify surgical patients for their individual risks [13].

Epidural analgesia can inhibit rehabilitation and hinder enhanced recovery after surgery (ERAS) protocols. There are several techniques that can be used to replace epidural analgesia, including, but not limited to, peripheral nerve blocks, catheter wound infusion, and other techniques of regional anesthesia [14]. Outpatient procedures may be a useful alternative, as they are associated with a reduction in postoperative morbidity [14]. In a meta-analysis of 170 randomized clinical trials of postoperative pain control following total knee arthroplasty (n = 12,530 patients), there were 17 different pain treatments, which were evaluated based on balanced pain scores, overall opioid consumption, and range of motion over the first 72 hours after surgery. In this study, epidural analgesia was inferior to most pain treatment regimens, while femoral/sciatic blocks were best for range-of-motion and optimal pain control [15]. ERAS protocols are increasingly coming under scrutiny. Before ERAS was implemented, the length of stay in the hospital associated with total knee replacement in the United Kingdom from 2008 to 2016 decreased from 5.8 days to 3.7 days, with a concomitant decrease in costs. After ERAS, the length of stay continued to decline but at a rate 50% slower with a similarly slowed rate of cost reduction [16]. A salient critique of ERAS is that its lack of individualization has rendered it obsolete in today’s surgical setting, where multimodal analgesia and regional anesthesia are common [17].

A growing body of evidence supports certain alternatives to epidural analgesia. A meta-analysis of 18 studies (n = 777) reported that paravertebral block was significantly superior to epidural analgesia in the first 24 hours after surgery in terms of reducing urinary retention rates, postoperative nausea and vomiting, and hypotension, but pain relief and the rate of pulmonary complications were similar over the first 48 hours after surgery [18]. A systematic review of 14 clinical trials of thoracotomy patients (n = 698) found paravertebral block provided similar pain control, length of stay, 30-day mortality, and rate of major complications as epidural analgesia, but paravertebral block was associated with fewer minor complications [19]. In meta-analyses of abdominal surgery studies, continuous wound infiltration was found to be as effective as epidural analgesia [20-22]. Transversus abdominis plane (TAP) block was found to produce analgesic results similar to those of epidural analgesia [23-25], and TAP block was associated with reduced opioid consumption in the first 24 hours after surgery [26]. A meta-analysis of 29 randomized controlled trials (n = 2,059) found preperitoneal wound catheters provided similar analgesic benefits after surgery as epidural analgesia, but opioid consumption, rate of hypotension, and patient satisfaction favored preperitoneal wound catheters [27]. A meta-analysis of 74 randomized clinical trials (n = 5,101) using 11 different colorectal surgery techniques found that spinal analgesia and TAP blocks offered superior balanced pain control and reduced postoperative opioid consumption compared to epidural analgesia in the first 24 hours after surgery [28]. However, regional approaches appear to be underutilized, even in orthopedic surgery [29].

Informed consent requires that patients be apprised of the risks as well as benefits of proposed medical treatments, including surgeries [30]. Yet, many patients seem unprepared for the postoperative experience. In a prospective study of 1,481 adult presurgical patients, 80% were unaware of the risk of CPSP, and 25% said this information might have changed their decision about surgery. In this survey, 78% who were informed about the risk of postoperative pain were informed by the surgeon, and none received this information from the anesthesiologist [31].

To reduce the barriers to better postoperative pain control, several specialties must converge, including surgery, anesthesiology, and acute pain services.

Surgery: When appropriate, surgeons should favor minimally invasive surgeries and employ nerve-sparing techniques. Unnecessary surgical interventions should be avoided.

Anesthesiology: Anesthesiologists should use regional anesthesia when appropriate, avoid ultra-short-acting opioids, deliver effective multimodal postoperative analgesia, and educate patients before surgery about what to expect in postoperative pain and the risk of CPSP.

Acute pain services: This service should screen for high-risk patients, follow patients after surgery, and make appropriate referrals to mental health, rehabilitation, or addiction specialists, when necessary.

Based on the Orebro acute pain services model, ward nurses can play an important role in pain management, including the administration of intravenous opioids and dose adjustments within prescribed limits, initiation of patient-controlled analgesia when appropriate, carrying outstanding orders for analgesic-associated adverse effects, and monitoring pain scores. There must be a collaboration between the surgeon and the anesthesiologist as well as the nursing staff for optimal results [32].

Multimodal Analgesia for Postsurgical Pain

Improving postoperative pain care should go beyond informed consent and education for patients and extend to organizational shifts in hospitals, making them more receptive to patient input, more accommodating to relevant new technologies, and prioritizing round-the-clock acute pain services [33]. Hospitals should emphasize the known value of multimodal or balanced analgesic protocols which use a combination of different pain relievers, anesthetics, and techniques to reduce pain with lower doses of drugs and fewer, less severe side effects [34]. While balanced analgesia is clearly an advancement, it requires a multidisciplinary approach, clinician training, good communication strategies with patients, and a dynamic protocol for pain control. In other words, balanced analgesia is beneficial, but can impose organizational challenges.

As pain, including acute postoperative pain, is multifactorial, it cannot always be adequately addressed by a single analgesic product with a single mechanism of action [35]. Pain is perceived by the brain, modulated by the central nervous system, transmitted through a signal system that utilizes the peripheral nervous system, and is transduced at the pain site, which can be the extreme periphery. The topical pain relievers, local anesthetics, and cyclo-oxygenase (COX) enzyme inhibitors that work well for interrupting pain transduction differ in mechanism from the opioids that influence pain perception and modulation. The α2-agonists that can contribute to reducing pain perception in the brain do not affect pain signal transmissions downstream. Thus, balanced analgesic approaches are required to suppress nociceptor activation and with that the perception of pain on multiple fronts [36-38].

Treating postoperative pain requires attention at all points of the patient’s journey, from preoperative education of the patient and risk assessments to intraoperative analgesic techniques, from postoperative anesthesia care unit protocols to pain control in the ward during recovery and prescription analgesics and instructions for home pain control upon patient discharge [36]. The World Health Organization’s analgesic ladder considers pain control from the dimension of pain intensity and recommends nonsteroidal anti-inflammatory drugs (NSAIDs) for mild pain, combination therapy of NSAIDs and a weak opioid for moderate pain, and NSAIDs plus a strong opioid for severe pain [39]. When necessary, adjunctive agents may be added, such as gabapentinoids, ketamine, lidocaine, or corticosteroids. Regional techniques may also be added. In addition, nonpharmacologic interventions may also be considered, such as physiotherapy, psychological interventions, and others. The selection of appropriate pharmacologic treatments must be based on evidence, such as the number-needed-to-treat (NNT) data, analgesic effects, ability to reduce inflammation, and the formulations available, as sometimes the route of administration plays a role in analgesic choices. Safety is a paramount consideration, as many analgesics are associated with side effects or adverse events, or may interact with other agents. When multimodal therapy is used, it is important to select appropriate drug combinations, that is, prescribing agents with beneficial synergistic interactions.

Prescribing choices for nonopioid agents include paracetamol (acetaminophen) and NSAIDs (e.g., dexketoprofen, ibuprofen) [40,41]. Opioid agents include tramadol, a weak opioid, and strong opioids, morphine and fentanyl. Adjunctive agents include ketamine, gabapentinoids, such as pregabalin or gabapentin, and lidocaine. Local anesthetics may also be helpful, such as bupivacaine and ropivacaine. Dexketoprofen trometamol is the S-(+) enantiomer of racemic ketoprofen and inhibits COX-1 and COX-2; the tromethamine salt enhances rapid absorption with rapid transit through the upper gastrointestinal tract. The onset of action for dexketoprofen tromethamine is about 30 minutes, faster than ketorolac, diclofenac, or tramadol [42]. Prescribers should also consider the NNT, which is specific to the agent, dose, protocol (single or multiple doses), patient population, comparator or control, and route of administration. While these factors can make head-to-head NNT comparisons difficult, dexketoprofen overall can be regarded as a safe, effective analgesic agent according to current standards and evidence [43].

NSAIDs are a broad class of agents and have been linked to bleeding risk, gastrointestinal side effects, and a risk for cardiovascular adverse events [44]. Dexketoprofen is of lower risk than many other NSAIDs and is a more powerful pain reliever than paracetamol alone [43,45]. The timing of dexketoprofen administration has been studied in several clinical trials. A clinical study comparing intravenous regimens of 50 mg dexketoprofen to 1 g paracetamol for postoperative pain control following lumbar disc procedures found that dexketoprofen offered significantly superior pain control in the first 24 hours after surgery, but morphine consumption was similar in both groups [46]. A study of 60 arthroscopic shoulder surgery patients evaluated the use of pre-emptive intravenous dexketoprofen compared to standard treatment and found pre-emptive dexketoprofen resulted in significantly lower visual analog pain scores and significantly longer sensory block time compared to standard care [47]. A study of 120 hip or knee arthroplasties randomized patients to receive two perioperative injections of either dexketoprofen 50 mg, lornoxicam 8 mg, or saline; following surgery, patients received intravenous patient-controlled morphine. Pain scores were lower in active treatment arms and lowest for dexketoprofen. Patients in active treatment arms consumed significantly less morphine after surgery, with the least morphine consumption in the dexketoprofen group [48]. A literature review reported that dexketoprofen trometamol had similar efficacy as a pain reliever to COX-2 inhibitors and offered a rapid onset of action and good tolerability [42].

Similar to NSAIDs, opioids are a broad class of drugs. Tramadol’s dual mechanisms of action make it a unique, even atypical, opioid analgesic [49]. Tramadol is a weak opioid with an affinity for the µ-opioid receptor and inhibits the reuptake of serotonin and norepinephrine. While tramadol is associated, in general, with milder and less frequent opioid-associated side effects and has less potential for abuse, the so-called “serotonin syndrome” and seizures may occur with supratherapeutic prolonged exposure [50]. Serotonin syndrome, caused by accumulating serotonin in the system, can be triggered by any number of drugs, including tramadol. Symptoms may include tremors, hyperthermia, shock, agitation, disorientation, tachycardia, vomiting, and others, and can be treated in many cases by discontinuation of the drug and supportive care [51]. The actual incidence of serotonin syndrome related to tramadol is unknown but is likely modest; many mild cases may resolve on their own. However, serotonin syndrome may have an abrupt onset and is potentially life-threatening.

In arriving at multimodal postoperative pain management regimens, it is important to properly combine drugs to achieve maximum benefit with minimal adverse events (see Table 1).

**Table 1:** Possible postoperative combination analgesic regimens. The efficacy and side effect values show whether the combination offers more or less than either agent used as monotherapy. APAP: paracetamol (acetaminophen); NSAID: nonsteroidal anti-inflammatory drug

Combination	Compared to monotherapy		Recommendation	Comments
	Efficacy?	Side effects?		
NSAIDs + NSAIDs	Same	Worse	No	Ceiling effect
APAP + opioids	Better	Similar or less	Yes	Use the lowest possible effective dose of opioids
NSAIDs + opioids	Better	Similar or less	Yes	
NSAIDs + adjuvant agents	Unclear	Unclear	Unclear	Depends on the patient and agents

When combining drugs for multimodal postoperative analgesia, it is important to consider that they have complementary pharmacokinetics and different mechanisms of action. Combination products that come in fixed-dose oral formulations are particularly helpful as they offer a balanced and synergistic pair of agents in a single pill, reducing the pill burden [35,37,52]. For control of mild-to-moderate postoperative pain, fixed-dose combination products of paracetamol/tramadol in 325/37.5 mg and 650/75 mg doses may be helpful. For moderate-to-severe postoperative pain, dexketoprofen/tramadol 25/75 mg or celecoxib/tramadol 200/75 mg are available. Other combinations are paracetamol/codeine, ibuprofen/codeine, and ibuprofen/oxycodone for mild-to-moderate pain and diclofenac/tramadol and ketorolac/tramadol for moderate-to-severe pain.

Dexketoprofen/tramadol may be particularly suitable for postoperative analgesia because it can be administered parenterally in the form of dexketoprofen 50 mg every eight hours together with tramadol 50-100 mg every six hours, allowing for a transition to dexketoprofen/tramadol 25/75 mg for eight to 12 hours, and finally to a fixed-dose combination oral product. The oral products would include dexketoprofen 25 mg and 50-100 mg of tramadol, either individually or in a fixed-dose single product [37].

The combination of dexketoprofen/tramadol 25/75 mg offers a lower NNT than other dose combinations of these two agents or than tramadol alone, dexketoprofen alone, or ibuprofen alone [53]. Dexketoprofen/tramadol has been shown to be safe and effective in treating pain following arthroplasty, visceral pain after hysterectomy, and other forms of acute pain [42,52,54-59].

Locoregional techniques can also be used for postoperative pain control, including brachial plexus blocks, nerve blocks, transverse nerve blocks, paravertebral blocks, epidural blocks, femoral blocks, adductor canal blocks, intravenous lidocaine infusions, and others. The type and location of these techniques depend on the surgical site and the individual patient. Nonpharmacologic interventions should not be omitted from postoperative analgesic regimens. This may include psychological interventions, physiotherapy, exercise, massage, music therapy, and laughter therapy [60]. For patients who respond to alternative approaches, meditation and acupuncture may provide relief. The multimodal approach can include pharmacologic treatments, locoregional techniques, and nonpharmacologic strategies.

Following the Guidelines for Managing Postoperative Pain

The American Pain Society (APS) together with the American Society of Anesthesiologists (ASA) and the American Society of Regional Anesthesia and Pain Medicine (ASRA) organized an interdisciplinary expert committee to develop an evidence-based guideline for postoperative pain management in adult and pediatric patients. Their findings were then approved by the American Society for Regional Anesthesia to create a clinical practice guideline published in 2016 [36]. In 2019, the United States Health and Human Services Pain Management Best Practices Inter-Agency Task Force set up a public-private partnership to address multidisciplinary approaches to perioperative pain control based on expert consensus [61]. Among the APS-ASA guidance are several core principles, including the use of multimodal postoperative analgesia that includes nonopioid pain relievers such as paracetamol (acetaminophen) and/or NSAIDs, the consideration of site-specific local anesthetic infiltration for surgical procedures, and preoperative oral celecoxib in appropriate adult patients.

A retrospective database study of 2,340,462 patients undergoing total hip or knee arthroplasty found 86.4% were administered multimodal postoperative analgesia, and those on multimodal regimens had significantly fewer respiratory and gastrointestinal adverse events, consumed significantly fewer opioids, and had a shorter length of stay compared to patients receiving opioid monotherapy [62]. NSAIDs have raised concerns about bone healing, limiting their use in fusions, healing fractures, osteotomies, and other procedures. In a study of 11 cohort and case-controlled studies, pooled odds ratio for nonunion when NSAIDs were administered was 3.0, but when only the higher-quality studies were analyzed, there was no significant association between NSAID exposure and nonunion [63]. Another retrospective analysis (n = 8,693) found that the short-term use of NSAIDs, defined as ≤3 weeks, was not associated with long-term complications with bone healing [64]. The role of NSAIDs has also been challenged in colorectal resection surgery as a potential driver of anastomotic leaks. In a retrospective database study (n = 2,756), 32% of patients received NSAIDs, either diclofenac or ibuprofen, for postoperative analgesia. In an unadjusted analysis, more diclofenac patients (7.8%) and ibuprofen (3.2%) patients had anastomotic leakage than controls, but multivariate logistic regression analyses showed that while diclofenac was a risk (7.2 odds ratio, p < 0.001), ibuprofen was not [65]. Thus, COX-2 inhibitors such as diclofenac should be used only with caution, if at all, following a colorectal resection procedure with a primary anastomosis, but the evidence is equivocal.

The safest, most effective, and best tolerated multimodal analgesic combinations depend on the type of surgery, the patient, and likely other factors such as the duration of the procedure and patient comorbidities. Most multimodal combination regimens are based on an opioid/nonopioid analgesic combination, whereby the nonopioid pain reliever may be an NSAID or paracetamol (acetaminophen). A gabapentinoid may be added. In some cases, intravenous ketamine or lidocaine may be appropriate in the immediate inpatient postoperative period [36]. Opioids as well as NSAIDs should be administered at the lowest effective dose for the shortest effective period of time.

The postoperative analgesic strategy should commence before the operation with a physical and psychological assessment of the patient, including current medications, comorbidities, chronic painful conditions, and determining if the patient has a history or active substance use disorder. Prior surgical history and response to pain medications can also be helpful. The patient’s pain levels should be assessed at baseline and throughout treatment using a validated instrument, such as a visual analog scale. Multimodal analgesic regimens are preferred [36], and patients and families should be educated about pain management. If the patient is taking opioid analgesics, the patient and family should be informed about the risks and benefits. Using a shared decision-making model, the clinician and patient should develop goals for postoperative pain management and functional recovery/rehabilitation [66]. As the patient heals, medications should be discontinued and/or tapered under clinical supervision. Many patients recover at home and take oral opioids. These patients should be given instructions on how to take these medications and how to dispose of unused oral opioids. If pain persists, pain levels should be assessed and analgesia re-evaluated. Mild-to-moderate postoperative pain in this phase may be better managed with physical therapy, exercise, or other nonpharmacologic means. If postoperative pain does not decrease as expected, if it is severe, or if the patient’s pain cannot be adequately controlled using reasonable means, refer the patient to a pain specialist.

The European Medicines Agency (EMA) has issued specific guidelines for pain control in children. Codeine is to be used with caution and only in those over age 12 when other nonopioid pain relievers are ineffective. Note that the EMA states codeine should not be used by patients under the age of 18 years who are undergoing tonsillectomy and/or adenoidectomy, as these patients are already at an elevated risk for respiratory complications [67]. The Food and Drug Administration has also issued a warning for the use of tramadol in children, as respiratory depression and death have occurred in children who received tramadol post-tonsillectomy and/or adenoidectomy and were ultra-rapid CYP2D6 metabolizers. Thus, in the United States, it is contraindicated in patients <12 years old, and in patients <18 years old post-tonsillectomy and/or adenoidectomy.

Pediatric Postoperative Pain Control

In the United States, 27% of pediatric patients in the hospital had moderate-to-severe pain, and the rates were the highest among teens (38%) and infants (32%) [68]. Geographic differences in perioperative and postoperative analgesia have been noted but are not yet fully elucidated, with many pediatric patients in developing nations receiving less than recommended doses [69]. Pain relief is recognized as a fundamental human right, which includes pediatric patients [70]. Yet, acute pain remains undertreated in the pediatric population.

Many myths surround pediatric pain management, even among clinicians. There is a debunked but long-standing myth that children are unable to feel pain. It is known that the neural pathways of pain perception function as early as 24 weeks of gestation [71]. Some clinicians still believe that infants and children are unable to reliably express pain. This is far from the truth, although children do not always express pain in the same way that adults do. Finally, another persistent myth is that even if children experience pain, they would never remember it. The fact is that children do remember pain and such memories can cause short-term physiologic disturbances and longer-term behavioral changes [72,73].

Other barriers to effective treatment of acute postoperative pain in pediatric patients involve deficits in clinician training. Physicians and nurses often underestimate pain in infants and overestimate the risks of opioids [74]. Prescribers may not feel confident prescribing strong pain relievers to children. In a survey of Canadian medical students, 87.6% of first- and second-year medical students and 75.0% of third- and fourth-year students said they were “uncomfortable” with pediatric pain management [75].

Pediatric pain assessment can be very challenging (see Table 2) but is foundational for effective pain control. When possible, pediatric patients should be asked to describe pain characteristics, localize pain site(s), and explain if the pain gets better or worse with certain movements.

**Table 2:** Challenges in assessing pediatric pain are numerous but there are strategies to help clinicians more reliably measure this pain, allowing for enhanced treatment.

Potential pitfall: Patient is…	Countermeasures	Comments
Preverbal, nonverbal	Using nociceptive stimuli, ascertain facial expressions, movements, or sounds that correlate with pain	Facial expressions, movements, postures, and verbalizations may correlate to pain intensities
Cognitively impaired		
Distressed with an unknown cause	Consider separation anxiety, fear, hunger, discomfort	Differential diagnosis
Refusing to talk about suspected pain	May fear that admitting pain will lead to injection or other unpleasant treatment	Rely on other signs (facial expressions, movements, sounds) to assess pain. Build trust with the child
	May fear that admitting pain will prolong the hospital stay	
	May have a culturally inspired or familial attitude that complaining about pain is a sign of weakness or not being “brave”	Encourage the child to talk about the pain in a noncomplaining “brave” way
Unable to describe the pain	Suggest terms (“burning” or “deep” or “cramps”) and ask about pain locations	“Normalize” the discussion of pain so the patient does not feel that he/she is an extreme case

Pain assessment for children should utilize a validated assessment tool such as the Wong-Baker FACES scale, which is helpful for children between the ages of five and 12 but can sometimes be used for even younger patients [76]. Whichever pain scale is used, this method should transpose to a quantifiable scale that will facilitate in-clinic decisions.

Analgesic options for postoperative pediatric patients include nonopioid pain relievers, opioids, and regional anesthesia techniques. The appropriate choice is based on the individual patient and clinical judgment. NSAIDs and paracetamol can be effective and are available for different routes of administration, but there is a ceiling effect to their effectiveness. Mu-opioid receptor agonists, such as morphine, can be used for postoperative pain control in children but should be used at the lowest effective doses for the shortest period of time. Mixed agonist antagonists, such as nalbuphine, act as agonists at some receptors and antagonists at others. In some cases, patient-controlled analgesia using morphine or fentanyl may be appropriate. For pediatric patients, pain pump control may be assigned to a nurse or a caregiver if the child is not able to manage the device.

There has been a general reluctance on the part of some clinicians to prescribe morphine or other opioids to pediatric patients [77]. Further research in pediatric and, in particular neonatal, pharmacokinetics is needed [78]. The active metabolite of morphine is morphine-6-glucuronide (M6G), eliminated via the renal system, and there can be a delay between the temporal course of M6G plasma concentration and its effects, resulting in a half-life delay that can exceed six hours, while morphine itself has a half-life of 2.8 hours. Note that M6G crosses the blood-brain barrier slowly [79]. In preterm infants, the half-life of morphine can be six to 12 hours but is variable and inversely related to gestational age. Babies experience a more rapid half-life; in children between the ages of one and six years, morphine has a half-life of about one hour. Newborns experience a much longer morphine half-life which may be attributed to M6G. It is believed that newborns have an underdeveloped opioid receptor system and metabolize M6G with difficulty. Addiction has not been observed in infants exposed to morphine. When prescribing morphine to infants, it is important to allow for slow drug clearance and to be alert to signs of drug accumulation, which can lead to respiratory depression [80]. Thus, opioid administration to preterm infants and neonates should occur only under close clinical supervision in a monitored setting.

Codeine, often combined with paracetamol, is considered a “weak opioid” and is sometimes prescribed for children and adults. About 10% of codeine is metabolized into morphine by way of the CYP2D6 enzyme, accounting for its analgesic benefits. However, genetics influence codeine metabolism. Approximately 10% of the population does not metabolize codeine properly into morphine, so the agent has little therapeutic effect. Another subpopulation is the “rapid metabolizer,” for whom even therapeutic doses of codeine can be life-threatening [81]. Breastfed infants with a genetic predisposition toward rapid metabolizing can be placed at serious risk when the mother is taking codeine [82].

Tramadol is a centrally acting analgesic, which is structurally a synthetic analog of codeine and is associated with fewer of the classic opioid-associated side effects. It is available in oral or intravenous formulations and is dosed at about 1 to 2 mg/kg in children [83]. Like codeine, it is also a pro-drug and needs to be metabolized by the CYP2D6 system to O-desmethyltramadol to be effective. Here lies the concern with the use of tramadol in the pediatric population, as noted above. However, a meta-analysis reported that evidence favoring the use of tramadol for pediatric postoperative pain management is low and to be viewed with caution [84]. Based on available research and evidence, pain management for children following surgery should emphasize nonopioid analgesics first, followed by opioids, and prefer regional anesthesia techniques when indicated.

The role of regional anesthesia in controlling postoperative pain in children has increased markedly since the 1980s. In adults, regional anesthesia has supplanted general anesthesia in some cases, but for children, regional anesthesia is performed under general anesthesia or sedation. Peripheral nerve blocks are associated with very low rates of morbidity. Transcutaneous stimulation and ultrasound-guided interventional techniques have facilitated nerve identification and plexus localization in children [85,86]. Echo-guided nerve blocks in the upper and lower extremities may actually be easier in children than adults. Small blocks can be highly effective in children, such as might be needed for circumcision, umbilical hernia, and hypospadias surgery [87,88].

More and more, spinal anesthesia is being used in infants to avoid general anesthesia, which is associated with potential neurocognitive side effects and respiratory complications. With the use of bupivacaine 0.5% (1 mg/kg up to 7 mg) plus clonidine 1 µg/kg with or without epinephrine, a study of infants undergoing surgery was successful in 89% of patients and provided anesthesia appropriate for procedures lasting from 60 to 100 minutes [89]. The main concerns for the use of spinal anesthesia in babies are lack of residual analgesia, short duration of action, and other risks associated with spinal anesthesia in general [89]. In adults, bupivacaine is metabolized hepatically and the drug is bound by plasma proteins. Neonates and babies have reduced circulation to the liver and thus larger proportions of the local anesthetic go unmetabolized, meaning they remain active and accumulate in serum circulation. Infants have low levels of serum albumin and α1 acid glycoproteins, necessary for drug binding, also leading to larger proportions of unbound amide local anesthetics [90].

Prehabilitation in Perioperative Medicine

The neuroendocrine and the inflammatory-immune responses are the two primary forms of stress response to major surgery. As tissue injury triggers cytokine release, minimally invasive surgery and central neural blockade can inhibit cytokine production as a stress response, but general anesthesia has little to no influence on cytokine production. There is a normal metabolic response to surgery in the form of hypermetabolism and hypercatabolism, which converts hepatic glycogen to glucose, at the same time that proteolysis affects skeletal muscles and lipolysis recruits from fat reserves. These various energy sources are necessary for postsurgical tissue repair. The consequences of these multiple surgical responses are increased serum glucose levels, increased plasma fatty acids and amino acids, and protein catabolism [91]. Thus, even with an uncomplicated surgery, patients still experience a loss of functional capacity following surgery. In patients with a low functional capacity reserve, malnourished, or sarcopenic, this surgical stress response will not only cause a deterioration of functional capacity that could potentially lead to a loss of postoperative functional independence but will also impair the ability of the body to properly cope with this surgical stress response, potentially triggering the appearance of postoperative medical complications. There are several factors that are correlated with poor postoperative outcomes, and multimodal prehabilitation aims at optimizing them before surgery. The first step in this process is to identify all these modifiable factors [92]. Cardiopulmonary reserves can be a significant predictor of risk for morbidity in noncardiopulmonary thoracic or abdominal surgery [93]. Diminished functional capacity has been identified as a risk factor for all-cause mortality following intra-abdominal surgical procedures [94]. Frailty is an independent predictor of postsurgical complications, extended length of stay, and other postsurgical risks among geriatric patients [95,96]. Indeed, preoperative levels of physical fitness are important predictors of postoperative complications [97]. However, subjective assessments of physical fitness were not as useful as those made using the validated instrument of the Duke Activity Status Index questionnaire [98]. Functional capacity can serve as a predictor of surgical outcomes in terms of postoperative morbidity and mortality, hospital length of stay, recovery, quality of life, and the degree of individual independence [98-100].

**Figure 1:**
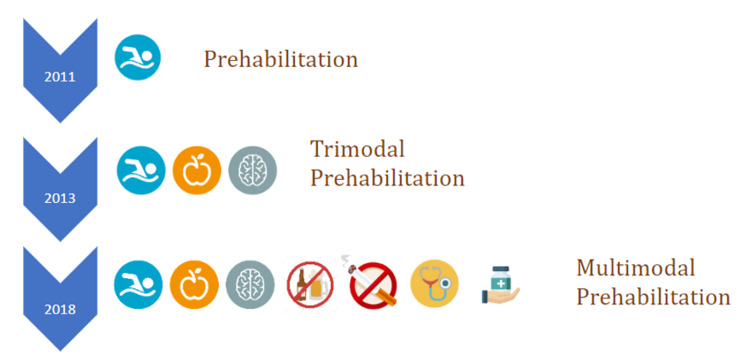
Prehabilitation paradigms from 2011 to 2018. Figure created by Miquel Coca Martinez.

The evaluation of patient fitness before surgery allows for a program to help improve patient health and fitness and reduce surgical risks [101]. By boosting functional capacity in advance of surgery, the patient will experience less lost function in the acute postsurgical period and will be able to rehabilitate with faster and better outcomes. Of course, the strategies behind prehabilitation have changed over the years, evolving into a multimodal paradigm (see Figure 1).

Prehabilitation protocols may be individualized to meet the specific needs and goals of the individual patient. Exercise regimens should vary in modalities and intensities based on the patient’s fitness level and specific needs. While some exercise programs rely on supervised instruction, other exercise protocols may allow for unsupervised exercise or hybrid programs. As a general rule, the exercise component of prehabilitation should consist of both aerobic exercise and strength training. This program can be supplemented with motivational interviewing, reflective listening, and recommendations to help patients overcome their personal barriers to better physical fitness. It may be helpful to encourage patients to increase their overall level of physical activity in daily life in addition to their exercise program.

Prehabilitation should include a nutritional component and a patient education intervention. The synergistic effects of exercise and nutrition to promote anabolism are well studied. Prehabilitation uses this synergistic effect to maximize the effect of exercise on functional capacity, as well as to promote an increase in muscle mass and function that will contribute to the body’s reserve before surgery. Emphasis on balanced meals with enough adequate protein intake and blood glucose management for diabetic patients are the bases for the nutritional component of prehabilitation.

Moreover, prehabilitation programs may incorporate psychosocial interventions, such as mindfulness meditation, deep-breathing techniques, and relaxation methods. Preparing for surgery is not just a physical endeavor, prehabilitation may also guide the patient to actively participate in the psychological preparation for the surgical journey. Patients should share in decision-making and be fully informed about the risks and benefits of their upcoming surgery [102]. The medical optimization of a presurgical patient can be challenging but may encompass smoking cessation, adjustments to pharmacologic regimens, and corrective protocols for anemia. In patients with chronic painful conditions, pain management should be optimized in advance of surgery.

All of these things necessitate a multidisciplinary team with good intercommunication. Depending on the patient’s condition and specific needs, this team may include a case manager, nutritional consultant, psychologist or counselor, physiotherapist, and anesthesiologist/surgeon team. Prehabilitation begins with a baseline assessment of patients, including their functional, nutritional, and psychosocial status plus an evaluation of their lifestyle, including current level of physical activity, smoking and drinking habits, and their living situation in terms of caregivers and support. This initial baseline assessment must also take into account the patient’s comorbid conditions.

Prehabilitation can improve perioperative function, postoperative recovery, and outcomes, even going beyond the immediate postoperative period [103]. Prehabilitation has been shown to be a cost-efficient intervention [103]. In a randomized, blinded controlled trial, aiming at high-risk patients awaiting elective major abdominal surgery, the prehabilitation group had significantly improved outcomes in terms of a 51% reduction in postsurgical complications and superior aerobic capacity after surgery compared to the control group [104]. In a secondary analysis of a randomized clinical trial, it was found that prehabilitation reduced the 30-day readmission rate, resulted in improved physical endurance at three and six months, and cost an average of 389 euros per patient [105]. Another recent multicenter randomized controlled trial with 251 participants found similar results in the reduction of medical postoperative complications [106]. Further, there is evidence it may improve tumor regression and colorectal cancer surgery outcomes [107,108].

There are often barriers to mainstreaming prehabilitation in clinical practice, although prehabilitation can be beneficial to the hospital. A prospective study in a single tertiary care hospital found in a per-protocol analysis that patients who completed prehabilitation programs had a significantly reduced mean length of stay, and those who underwent the most aggressive surgeries had a significant reduction in the length of stay in the intensive care unit. The mean cost savings generated per patient with prehabilitation versus controls was 3,093 euros [109]. Implementing these programs requires a patient-centered, preoperative process that is equipped to manage multi-morbidities, effectively addresses unhealthful habits, and improves the physical, nutritional, and psychological status of the preoperative patient [109]. This study found that prehabilitation was most effective in patients who were to undergo aggressive surgical interventions and who completed the entire prehabilitation protocol. About two-thirds of the patients (66%) in this study did not complete the prehabilitation program, which was attributed to unrealistically high standards for participation (80% attendance over a four-week program), transportation problems, or other logistical issues on the part of patients and their families, and patients who had other surgical or medical interventions that interfered with the prehabilitation program [109]. This led to five recommendations that may help advance prehabilitation programs in a clinic (see Table 3) [107].

**Table 3:** Proposals and strategies to incorporate prehabilitation in an existing surgical center.

Proposal	Tactics	Comments
Increase the rate of completion of the prehabilitation program	Improve program accessibility	Patients sometimes had logistical or scheduling conflicts that impeded full participation; these barriers should be removed as much as possible
	Align the program with surgical and medical agendas	
	Promote patient engagement and self-efficacy	
Refine and standardize the service delivery	Redesign the program to use a lean approach	Create different tiers of patients to help individualize approaches but do not allow this to complicate the program
	Use a three-layer approach (low, medium, and high risk) and individualize to each patient	
Better risk assessment	Use multilevel predictive models	Validated risk assessments may be vital and prehabilitation programs may require individualization to meet the needs of each patient
	Personalize the interventions for each patient	
Digital support	Cloud-based mature digital support to ensure stakeholders’ interoperability	Prehabilitation must be integrated into the clinical workflow and process at all levels, including into the digital landscape
	Broaden the scope of current digital efforts to include prehabilitation	
Community-based interventions	Transfer services, when possible, to community-based stakeholders such as recreation or sports centers	Many prehabilitation activities are suitable for community-based centers, such as recreation facilities, which can be recruited as partners
	Provide remote support for home-based activities	
	Encourage networking among various healthcare tiers to promote better collaboration	

In this connection, it is important to recognize that surgical risks vary from low to moderate to high and these risk strata may necessitate somewhat different approaches (see Table 4).

**Table 4:** Varying prehabilitation programs to meet the needs of low-risk, moderate-risk, and high-risk patients [109].

	Low risk	Moderate risk	High risk
Promotion of physical activity	Yes	Yes	Yes
Supervised exercise training	Exercise tips and recommendations	Community-based programs or home-based programs	Hospital
Nutrition	Advice and general tips	Targeted advice	Individualized program
Psychological support	Mindfulness exercises and general tips	Mindfulness exercises and group-based sessions	Individualized or targeted programs

Discussion

Despite the marked decrease in nonessential surgeries during the pandemic, the rate of surgeries around the world has rebounded and will likely continue to increase [110]. In the United States, over 19 million ambulatory surgical procedures were performed in 2018 [111]. Globally, over 300 million surgeries were performed in 2012, but despite these burgeoning numbers, surgical morbidity remains a persistent challenge [112]. About 8 million people around the world die each year following major surgery and about double that experience postoperative complications [112], sometimes detectable at an early stage with the study of inflammatory markers [113]. One of the adverse events following surgery is uncontrolled or undertreated postsurgical pain which sometimes transitions into chronic postsurgical pain. The mechanisms involved in this transition and risk factors for chronic postsurgical pain remain to be more fully elucidated, but the adequate treatment of acute postsurgical pain is a clinical imperative [11]. While much remains to be better studied and explored, the tools are there to manage postoperative pain-but the will may not be.

Specific recommendations for postoperative pain control regimens exceed the scope of this work but vary depending on surgical as well as patient factors. The surgical factors include the type of surgery, how invasive is it, surgical duration, and perioperative pain management. Patient factors include the patient’s overall health, frailty, comorbid conditions, and psychological factors such as catastrophizing. Effective and safe pain control following surgery is possible using a multidisciplinary approach with balanced analgesia.

## Conclusions

Acute postsurgical pain is prevalent and often undertreated despite our analgesic armamentarium. A surprisingly high proportion of acute postsurgical pain can transition to chronic postsurgical pain, which is not only challenging to treat but also exposes patients to pain that may last for a minimum of two to three years, and in others for the rest of their lives. Balanced or multimodal analgesia can be beneficial in that it addresses multimechanistic pain with agents with different and ideally synergistic mechanisms of action. Dexketoprofen and tramadol combinations have been demonstrated safe and effective for managing many forms of acute postsurgical pain, but analgesic regimens must be tailored to meet the individual needs of the patient. Analgesic choices should be based on guidelines and evidence but combined, sometimes with nonpharmacologic means or prehabilitation strategies, to personalize analgesic strategies. Further study is needed for treating postoperative pain in general and in specific special groups, such as the neonatal and pediatric populations.
